# Editorial: Robotics for smart farms

**DOI:** 10.3389/frobt.2022.1113440

**Published:** 2023-01-04

**Authors:** Luis Emmi, Roemi Fernandez, José Miguel Guerrero

**Affiliations:** ^1^ Centre for Automation and Robotics (UPM-CSIC), Madrid, Spain; ^2^ Department of Signal Theory and Communications, Telematics and Computing, Rey Juan Carlos University, Madrid, Spain

**Keywords:** smart farms, robotics, precision agriculture (PA), farm automation, AI in agriculture

Farming is facing many economic challenges in terms of productivity, cost-effectiveness and labor shortage (Meuwissen et al., 2019). Farming is approaching into the “smart” era, where operations become digitalized and data-driven, making possible to monitor and control many diversified and interconnected procedures such as crop growth assessment, pest control, disease monitoring, irrigation, planting, crop spraying, field analysis, among others (Klerkx et al., 2019). This approach will be carried out through an increasing number of automated devices and tools interacting with the need to track the production processes for operations optimization or traceability toward consumers. The introduction of automation, including intelligent robots, automated sensing, modeling, decision-making, artificial intelligence (AI), smart analyses, planning and actuation, and the role of genotypes *versus* the environment, allows the agricultural operations to be enhanced efficiency (Gonzalez-de-Santos et al., 2020). Additionally, improved actuation, sensing and monitoring of the production reduce the environmental impact, increase quality, quantity, and sustainability. Robotics for smart farms gives a unique possibility to assess and evaluate agricultural sustainability with an unprecedented level of accuracy and precision.

This Research Topic provides a useful overview of the capabilities and the applications of robotic systems (both aerial and ground robots) and human-robot collaboration in combination with artificial intelligence (AI) strategies for the development of different tasks in agricultural environments.

The applications in the current Research Topic focus on the use and implementation of robotic systems in various agricultural tasks. For example, Ulloa et al. propose an optical sensory system for data acquisition and processing in precision agriculture for Robotic Fertilization process. They integrate different sensory systems such as a LIDAR, an RGB and a multispectral camera, as well as a robotic arm equipped with a fertilization system in a robotic platform to reconstruct rows crops and extract information for fertilization with the robotic arm.

Moreover, in recent years site-specific sprayer solutions (including pesticides and fertilizers) applied by the use of unmanned aerial vehicles (UAVs) have gained interest in the agricultural sector. Ma et al. present a data-driven framework to predict density distribution patterns of vermiculite dispensed from a hovering unmanned aerial vehicle (UAV) as a function of UAV’s movement state, wind condition, and dispenser setting. The authors propose a model based on the UAV altitude, wind speed, wind direction, and the dispenser settings (aperture size and flow rate) to estimate the two-dimensional distribution of low-density materials as the vermiculite, which is often used as carrier in UAV-based distributions of biological control agents.

An important element in the introduction of robotic systems in smart farming is the cooperation between operators and robots. Harman and Sklar propose a multi-agent task allocation method that assists farm managers and team leaders to manage the harvesting workforce effectively and efficiently. The authors propose a variation of Round Robin (RR) for the problem of assigning workers to fields, and market-based task allocation mechanisms are applied to the challenge of assigning tasks to workers within the fields. The authors define a worker model, learned from observing each worker’s performance during the harvesting season, that can be a human or a robot, hence anticipating the methodology adoption for human-robot workforces.

One of the great challenges that robotic and AI systems currently face is the lack of a regulatory framework and guidelines that can guarantee safety and operability in agricultural production systems. For this Eastwood et al. propose a “responsibility by design” approach to innovation in robotics and automation, using the development of batch robotic milking in pasture-grazed dairy farming as a case study. The authors highlight wider considerations that technology developers and policymakers need to consider when envisaging future innovation trajectories for robotics in smart farming, including the impact on work design, worker wellbeing and safety, changes to farming systems, and the influences of market and regulatory constraints.

This Research Topic shows a small but important glimpse of what robotics and artificial intelligence can contribute to agricultural production systems, as a means of managing agricultural land, animals, and people more effectively, efficiently, and automated. The approaches presented in this Research Topic involve the use of sensors, machines, drones, and satellites to monitor animals, soil, water, and plants. The data obtained through monitoring are used to interpret the past and predict the future, with the aim of making more timely or accurate decisions, both on-farm and in the supply chain.
